# Influence of polyvinyl chloride (PVC) microplastic morphology on bacterial survival during thermal and ultraviolet-C inactivation in aqueous systems

**DOI:** 10.1128/aem.01025-26

**Published:** 2026-08-28

**Authors:** Sungjun Hwang, Wenjing Lu, Xu Yang, Kang Huang

**Affiliations:** 1 Department of Biological Systems Engineering, Washington State University 6760, Pullman, Washington, USA; 2 Department of Nutrition and Food Science, California State Polytechnic University 7173, Pomona, California, USA; University of Georgia Center for Food Safety, Griffin, Georgia, USA

**Keywords:** microplastics, polyvinyl chloride, thermal processing, UV-C, bacterial inactivation, foodborne pathogens

## Abstract

Microplastics are increasingly detected in water and food-associated environments, yet how particle morphology influences microbial persistence during inactivation remains poorly understood. This study aims to develop polyvinyl chloride (PVC) microplastic particles with distinct morphologies and controlled size ranges, and to evaluate how particle morphology influences bacterial attachment and survival during thermal and ultraviolet-C (UVC) inactivation in aqueous systems. First, we developed a PVC microplastic model system with distinct morphologies and controlled size fractions. Original PVC particles were relatively smooth and rounded, whereas irregular particles were generated by compounding, pelletizing, and cryogenically grinding the same PVC material, followed by sieving into defined size ranges. Second, using this model system, we evaluated how PVC particle morphology affected bacterial attachment and survival during thermal and UVC inactivation in aqueous systems. *Escherichia coli* O157:H7 and *Listeria innocua* were used as model gram-negative and gram-positive bacteria. Confocal microscopy showed attachment of *E. coli* to both particle types, with stronger association on ground PVC particles. In the presence of PVC particles, thermal log reductions decreased to approximately 4.0–5.1 log CFUs/mL, compared with about 5.9 log CFU/mL in microplastic-free controls, while UVC log reductions decreased to about 3.9–4.6 log CFU/mL, compared with about 5.2–6.0 log CFU/mL in microplastic-free controls. Ground PVC provided greater protection during thermal treatment, whereas both particle types similarly reduced UVC inactivation. These findings show that PVC microplastics can interfere with microbial control processes and highlight particle morphology as an important factor influencing bacterial survival in aqueous environments.

**IMPORTANCE:**

Microplastics are increasingly present in water and food-related environments, but their effects on microbial control processes remain poorly understood. This study demonstrates that PVC microplastics can reduce the effectiveness of both thermal and UVC inactivation of foodborne pathogens in aqueous systems. By developing PVC particles with distinct and defined morphologies, we further demonstrate that particle shape and surface characteristics influence bacterial attachment and survival, particularly during thermal treatment. These findings provide new mechanistic insight into how microplastics may alter bacterial persistence under disinfection or food-processing conditions. This work highlights microplastic morphology as a previously underappreciated factor affecting microbial inactivation and establishes a useful PVC microplastic model system for future studies on microplastic–microbe interactions in environmental and food-associated settings.

## INTRODUCTION

Microplastics, defined as plastic particles smaller than 5 mm, have raised growing concern in environmental and food-related research due to their persistence and widespread occurrence in water and food systems (1). Microplastics originate from both primary sources, such as industrial microbeads, and secondary sources, including the fragmentation of larger plastic materials such as packaging films and consumer products (2). Once released into the environment, microplastics can spread through aquatic systems, enter drinking water supplies (3), and subsequently reach food systems through multiple routes, including contaminated processing water and bioaccumulation in aquatic organisms (4). Their detection in products such as seafood, salt, honey, milk, and drinking water highlights the growing relevance of microplastics to food safety (5).

Among common plastic materials, polyvinyl chloride (PVC) is widely used in industrial and consumer applications, such as pipes, construction materials, packaging, and some food-contact materials due to its durability, chemical resistance, and relatively low cost (6, 7). However, its widespread use leads to the formation of secondary PVC microplastics through processes, including mechanical abrasion, material degradation, and fragmentation of plastic waste (8, 9). Consequently, PVC microplastics have been detected in aquatic environments, sediments, and drinking water systems (10, 11). In addition, PVC often contains additives such as plasticizers and stabilizers that may be released during degradation (12, 13), further increasing concerns regarding potential environmental and health effects. Despite this relevance, PVC remains less studied than polymers such as polyethylene and polystyrene in microplastic research.

A persistent limitation of microplastic-related research is the lack of model microplastic particles with well-defined and comparable properties (14). Many previous studies have used commercially available beads or irregular fragments generated by grinding or environmental collection, which often differ simultaneously in polymer type, size, shape, and surface characteristics (15). This variability makes it difficult to isolate the contribution of a single factor, such as particle morphology, to microbial attachment or treatment resistance. The problem is especially evident for PVC, for which relatively few model particles with controlled morphology and size are available. Therefore, the development and characterization of PVC microplastics with defined surface features and comparable size distributions can provide a useful platform for mechanistic studies and represent an important contribution to the field.

Microbial inactivation is central to food safety, and thermal treatment and ultraviolet-C (UVC) irradiation are widely used to control microbial contamination in aqueous and food processing systems. Thermal treatment inactivates microorganisms through protein denaturation, membrane disruption, and nucleic acid damage, and its effectiveness depends on factors, including temperature and treatment time (16). UVC irradiation is frequently used for microbial control in juices, beverages, and other liquid food systems (17); it causes photochemical DNA damage that inhibits replication while generally having minimal impact on food quality compared with thermal treatment (18). The effectiveness of these treatments depends on direct microbial exposure to the inactivation condition. Any factor that reduces such exposure, including shielding or surface-associated protection, may decrease treatment efficacy.

Most current studies have focused on microplastic transport, environmental distribution, or microbial colonization of plastic debris in aquatic environments. For instance, microplastics can alter microorganism behavior during treatment by serving as attachment sites and protective substrates (19, 20). Surface properties, such as roughness, hydrophobicity, and surface charge, have been shown to influence bacterial colonization and biofilm formation (21). Similarly, microplastics can significantly alter the transport and deposition behavior of bacteria in porous media by modifying retention sites, surface interactions, and physical entrapment mechanisms. A study also noted that microplastics can act as protective substrates during disinfection processes by reducing ultraviolet transmittance, consuming disinfectants, and shielding microorganisms from exposure, decreasing the efficiency of ultraviolet and chemical disinfection in water and wastewater systems (22). However, the role of microplastic morphology in bacterial survival during food-related sterilization processes remains poorly understood.

This study aims to develop PVC microplastic particles with distinct morphologies and controlled size ranges, and to evaluate how particle morphology influences bacterial attachment and survival during thermal and UVC inactivation in aqueous systems. First, we established a PVC microplastic model system with well-defined particle morphologies. This was achieved by preparing (i) original PVC particles with relatively smooth surfaces and (ii) mechanically ground PVC particles exhibiting more irregular and rough surface features. Both particle types were subjected to the same sieving procedure to ensure a comparable sieve-defined size range. This approach provides a set of PVC microplastics with controlled and distinct morphologies, enabling systematic mechanistic investigations. Second, using this model system, we investigated how microplastic particle morphology influences bacterial survival during thermal and UVC inactivation in aqueous systems. Gram-negative *Escherichia coli* and gram-positive *Listeria innocua* were selected as representative bacteria to capture potential differences in cell envelope structure and treatment response (23, 24). Particle morphology and size distribution were characterized by scanning electron microscopy and particle size analysis, while bacterial attachment was determined using confocal microscopy and plate counting methods, and bacterial survival after thermal and UVC treatment was quantified by colony-forming unit (CFU) analysis. Overall, these results not only clarify the role of PVC microplastic morphology in bacterial inactivation but also establish a defined PVC microplastic platform for future studies in food- and environmental-microbiology-related research and applications.

## MATERIALS AND METHODS

### Preparation of PVC microplastics with different morphologies

Polyvinyl chloride (SE-950, Shintech Inc., USA) was used as the model microplastic material. The as-received PVC particle exhibited relatively smooth surfaces, near-spherical shapes, and a low aspect ratio. To generate irregular particles with rough surfaces, the original PVC particles were first compounded using an internal mixer (QE-70C, ACS Materials, China). The compounded material was then pelletized using a granulator (SFS290049, HayWHNKN, China) equipped with a 6 mm screen. The resulting pellets were subsequently cryogenically ground using a cryomill (Cryomill, Retsch GmbH, Germany) equipped with eight 5 mm stainless steel grinding balls and a 25 mL stainless steel grinding jar. Approximately 6 mL of sample was loaded into the grinding jar. Cryogenic grinding was conducted for three cycles, each consisting of a precooling for 2 min at 5 s^−1^, grinding for 5 min at 28 s^−1^, and intermediate cooling for 40 s at 5 s^−1^. In this study, the as-received PVC particles and mechanically processed particles are referred to as original PVC and ground PVC particles, respectively.

Both original PVC and ground PVC particles were then size-fractionated using a sonic sifter (Gilsonic Autosiever Sonic Sifter, Gilson, USA). A stack of acrylic-frame stainless steel sieves with mesh sizes of 500, 180, 90, and 20 μm (GAA series, Gilson, USA), in accordance with ASTM E11, was used. The particle fraction passing through the 180 μm sieve and retained on the 90 μm sieve was used for subsequent analysis to ensure comparable particle size distributions between the two particle types. This size range falls within the range of microplastics detected in drinking water, where particles in the range of 50–150 μm have been reported in previous studies (10, 25). In addition, microplastic particles around 130 μm have been reported to translocate into human tissues, highlighting the potential biological relevance of this size range (4).

Microplastic suspensions were prepared by dispersing 100 mg of PVC particles (size range: 90–180 μm) in 50 mL of deionized water containing 0.088 wt.% Tween-20 (Sigma-Aldrich, USA), corresponding to a final microplastic concentration of 0.2 wt.%. Tween-20 was added to improve wetting and dispersion of the PVC particles, which were very light and tended to remain floating at the water surface. The mixture was then stirred for 10 min, followed by sonication using an ultrasonic processor (Q700, Qsonica, USA) operated at 20 kHz and 80% amplitude to further break up particle aggregates and facilitate particle settling and suspension in the aqueous phase. The resulting microplastic suspensions were then used for subsequent thermal and UVC inactivation experiments.

### Morphological analysis of PVC microplastics

The morphology of PVC microplastics was analyzed using a field-emission scanning electron microscope (FE-SEM, Apreo VolumeScope, Thermo Fisher Scientific, USA). Images were collected at an accelerating voltage of 5.0 kV. Prior to imaging, the samples were gold-coated using a sputter coater (Hummer 6.2, Anatech, USA) to improve electrical conductivity.

Particle aspect ratio was determined from SEM images using ImageJ software (Version 1.53e, National Institutes of Health, USA). The aspect ratio was defined as the ratio of the major axis to the minor axis of each particle. For each sample, at least 200 particles were analyzed.

Particle size distribution and average particle size were measured using a laser diffraction analyzer (Mastersizer 3000, Malvern Instruments, UK) equipped with a dry dispersion unit (Aero S, Malvern Instruments, UK). Approximately 1 g of sample was loaded into the dry dispersion unit, and a refractive index of 1.53 was applied for analysis (26). The average particle size was reported as the Sauter mean diameter D [3,2], as provided by the instrument. The D [3,2] value represents the surface area–weighted mean diameter and is defined in equation 1 (27).


(1) $$D\;\left[3,2\right]\text{=}\frac{\text{1}}{\sum_{i}\frac{P_{i}}{d_{i}}}\text{=}\frac{\text{6}}{\text{specific area}}$$

where *d*_*i*_ is the mean diameter of class *i*, and *P*_*i*_ is the relative volume probability of class *i*.

In addition to D [3,2], the characteristic percentiles D10, D50, and D90 were obtained from the cumulative volume-based particle size distribution. D10, D50, and D90 represent the particle diameters below which 10%, 50%, and 90% of the total sample volume are contained, respectively. D50 was used as the median particle diameter.

### Bacterial strains, media, and growth conditions

*Escherichia coli* O157:H7 (ATCC 700728) and *Listeria innocua* (ATCC 33090) were used as a model gram-negative foodborne pathogen and a surrogate for a gram-positive foodborne pathogen *Listeria monocytogenes*, respectively. Frozen stock cultures stored in liquid nitrogen were streaked onto agar plates and incubated overnight. Luria–Bertani agar and tryptic soy agar were used for plating of *E. coli* O157:H7 and *L. innocua*, respectively. Before each experiment, a single colony was picked from the agar plate and inoculated into the corresponding liquid medium, followed by incubation overnight. After incubation, the bacterial cultures were then centrifuged, washed twice with sterile phosphate-buffered saline (PBS), and resuspended in sterile PBS to a final concentration of approximately 10^9^ CFU/mL.

### Thermal and UVC treatments

Thermal inactivation was conducted in aqueous systems in the presence or absence of PVC microplastics. Control samples were prepared by mixing 100 μL of bacterial suspension (~10^7^ CFU/mL) with 100 μL of sterile PBS. Microplastic-containing samples were prepared by mixing 100 μL of bacterial suspension (~10^7^ CFU/mL) with 100 μL of 0.2 wt.% PVC suspension containing either original or ground PVC particles, followed by incubation at ambient temperature for 15 min to allow bacterial attachment to the microplastic surfaces. For treatment, 200 μL of each sample (bacterial suspension with or without microplastics) was added to 800 μL of preheated sterile PBS in a heating block, resulting in a final bacterial concentration of approximately 10^6^ CFU/mL. A final PVC concentration of 0.02 wt.% is also in the range used in prior aqueous disinfection studies examining microplastic interference with microbial inactivation (28). The treatment conditions were 67°C for 15 s for *E. coli* O157:H7 and 70°C for 15 s for *L. innocua*. These conditions were selected to provide mild but measurable inactivation without causing complete lethality, thereby allowing the effect of PVC microplastics on bacterial survival to be detected. Using temperatures slightly below or near this benchmark allowed us to create a sublethal thermal challenge appropriate for comparing treatment outcomes in the presence or absence of microplastics. This rationale is consistent with published work on commercial-type milk pasteurization and thermal inactivation studies of foodborne pathogens in the ~68–72 °C range (29, 30). After treatment, samples were immediately cooled in an ice bath. Each treated suspension was then serially diluted in PBS, and 100 μL of each dilution was plated for bacterial enumeration.

Ultraviolet-C irradiation was also performed in the presence or absence of PVC microplastics. Control samples were prepared by mixing 100 μL of bacterial suspension with 900 μL of sterile PBS. Microplastic-containing samples were prepared by mixing 100 μL of bacterial suspension (~10^7^ CFU/mL) with 100 μL of 0.2 wt.% PVC suspension containing either original or ground PVC particles and 800 μL of sterile PBS, followed by incubation at ambient temperature for 15 min to facilitate bacterial attachment. The samples were irradiated using a UV crosslinker (Fisherbrand, USA) equipped with a 253.7 nm UVC lamp (USHIO G8T5, Japan), with exposure times of 8 s for *E. coli* O157:H7 and 18 s for *L. innocua*. The UVC irradiance at the sample position was measured using a UVX radiometer (Analytik Jena, USA) equipped with a UVX-25 sensor probe (254 nm), which was approximately 11 mW/cm^2^. Based on this irradiance, the UVC doses were 88 mJ/cm^2^ and 198 mJ/cm^2^ for 8 s and 18 s, respectively. Following treatment, samples were collected and serially diluted for microbial analysis.

### Analysis of bacterial–microplastic interactions

To visualize and quantify bacterial attachment to PVC microplastics, *E. coli* O157:H7 was incubated with PVC particles under the same conditions used for the attachment assay. Briefly, *E. coli* cells were first stained with SYBR Green I (Invitrogen, Thermo Fisher Scientific, USA) by mixing 500 μL of bacterial suspension with 500 μL of SYBR Green I working solution (1:400 in PBS) and incubating at room temperature for 5 min. The stained cells were then washed with PBS to remove excess dye and resuspended in PBS. For the attachment assay, 100 μL of the stained bacterial suspension (~10^9^ CFU/mL) was mixed with 100 μL of 0.2 wt.% PVC suspension containing either original or ground PVC particles and incubated for 15 min to allow bacterial attachment. Subsequently, 800 μL of PBS was added to bring the total volume to 1 mL, resulting in the same final bacterial and microplastic concentrations used in the plate counting assay.

For confocal imaging, the incubated samples were washed with PBS to remove unbound cells, and the particle-associated cells were resuspended for imaging. Images were acquired using a confocal laser scanning microscope (Leica TCS SP8, Leica Microsystems, Germany). SYBR Green fluorescence was excited at 498 nm and detected at 508–650 nm. A transmission channel was collected simultaneously to obtain bright-field images. Z-stack images were collected for overlay and three-dimensional reconstruction using LAS X software (Leica Microsystems, Germany). All images were acquired using the same pixel size and spatial scale.

For quantitative analysis of bacterial attachment, the same particle–bacterial mixtures were filtered through a 200-mesh stainless-steel screen with an approximate pore size of 75 μm. The PVC particles retained on the screen, together with the associated bacteria, were recovered by rinsing the screen with 600 μL of PBS into a tube, followed by an additional 400 μL of PBS, resulting in a final recovery volume of 1 mL. The recovered samples were then vortexed, serially diluted, and spread-plated for bacterial enumeration.

### Statistical analysis

Statistical analysis was performed using the GraphPad Prism software V. 10.6.1 (GraphPad Software, Inc., La Jolla, CA). To compare the bacterial inactivation and attachment, one-way ANOVA followed by Tukey’s test was implemented. In all analyses, a 95% confidence interval was used.

## RESULTS AND DISCUSSION

### Production and characterization of PVC microplastics with distinct morphologies

**Fig 1:**
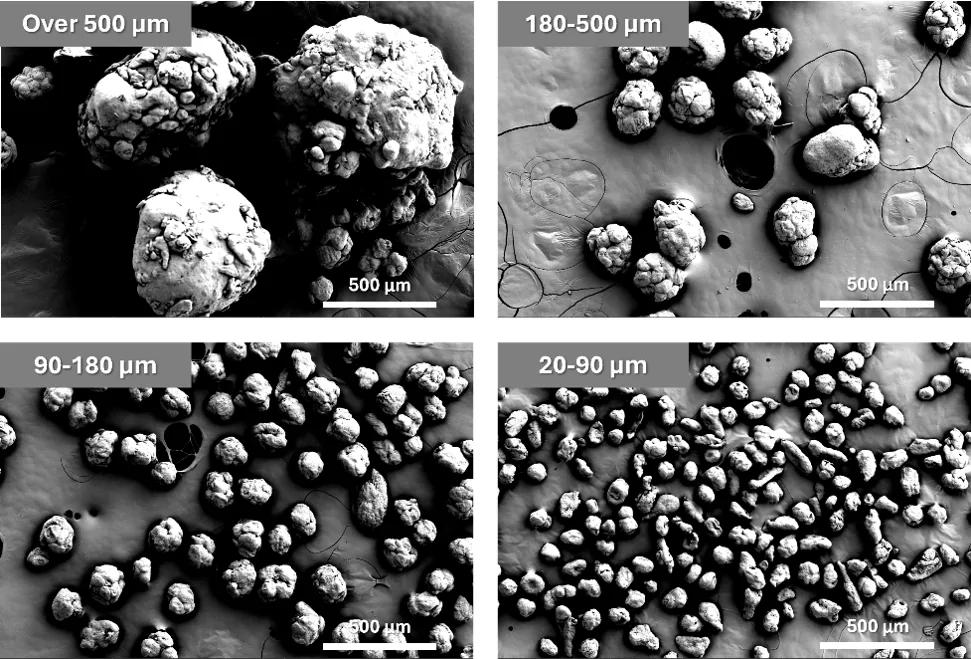
Representative SEM images of original PVC particles in different size fractions.

**Fig 2:**
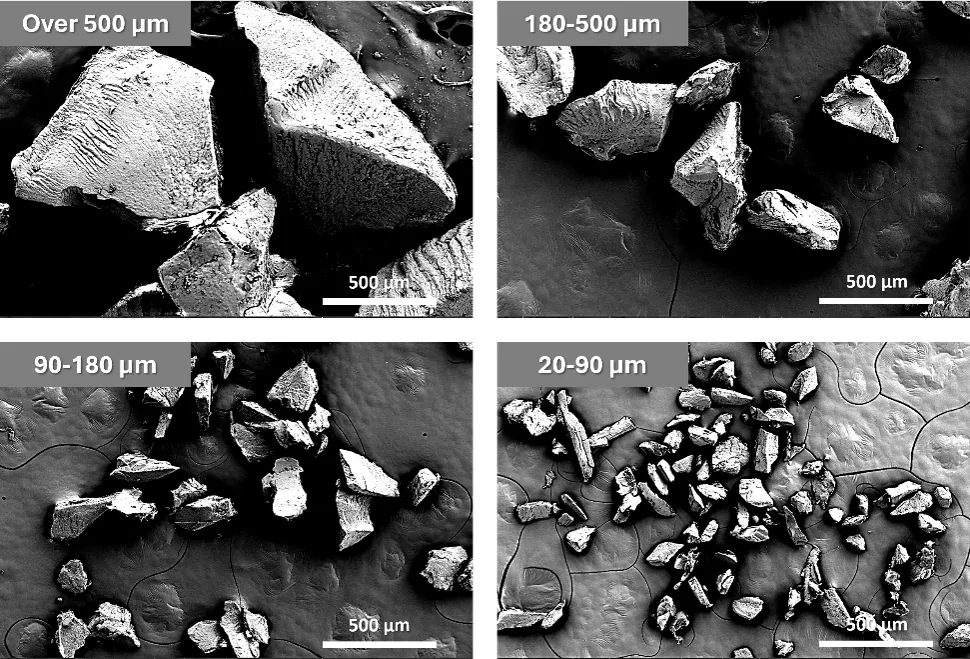
Representative SEM images of ground PVC particles in different size fractions.

A key objective of this study was to establish a PVC microplastic model system with clearly distinct particle morphologies while maintaining a comparable size range for subsequent bacterial interaction and inactivation experiments. As shown in Fig. 1, the original PVC particles exhibited relatively rounded surfaces and compact structures across all size fractions. In contrast, the ground PVC particles generated by cryogenic grinding displayed markedly different morphological features (Fig. 2), including more angular shapes, fractured surfaces, and elongated fragments. These differences indicate that cryogenic grinding effectively transformed the original PVC particles into irregular microplastic particles with substantially altered morphology. This morphological change is consistent with the brittle fracture behavior of PVC below its glass transition temperature, where repeated impact and shear during cryomilling generate fragmented particles with rougher and less uniform structures (31, 32).

The sieving process further enabled separation of both particle types into defined size fractions, including >500 µm, 180–500 μm, 90–180 μm, and 20–90 μm (Fig. 1 and 2). Across all fractions, the original PVC particles generally retained their rounded morphology, whereas the ground PVC particles consistently showed fractured and irregular features. This result indicates that particle morphology was primarily governed by the processing method rather than the size fraction itself. Such controlled preparation is important because many previous microplastic studies have relied on particles with less defined or overlapping physical characteristics, making it difficult to isolate the specific role of morphology (33, 34).

**Fig 3:**
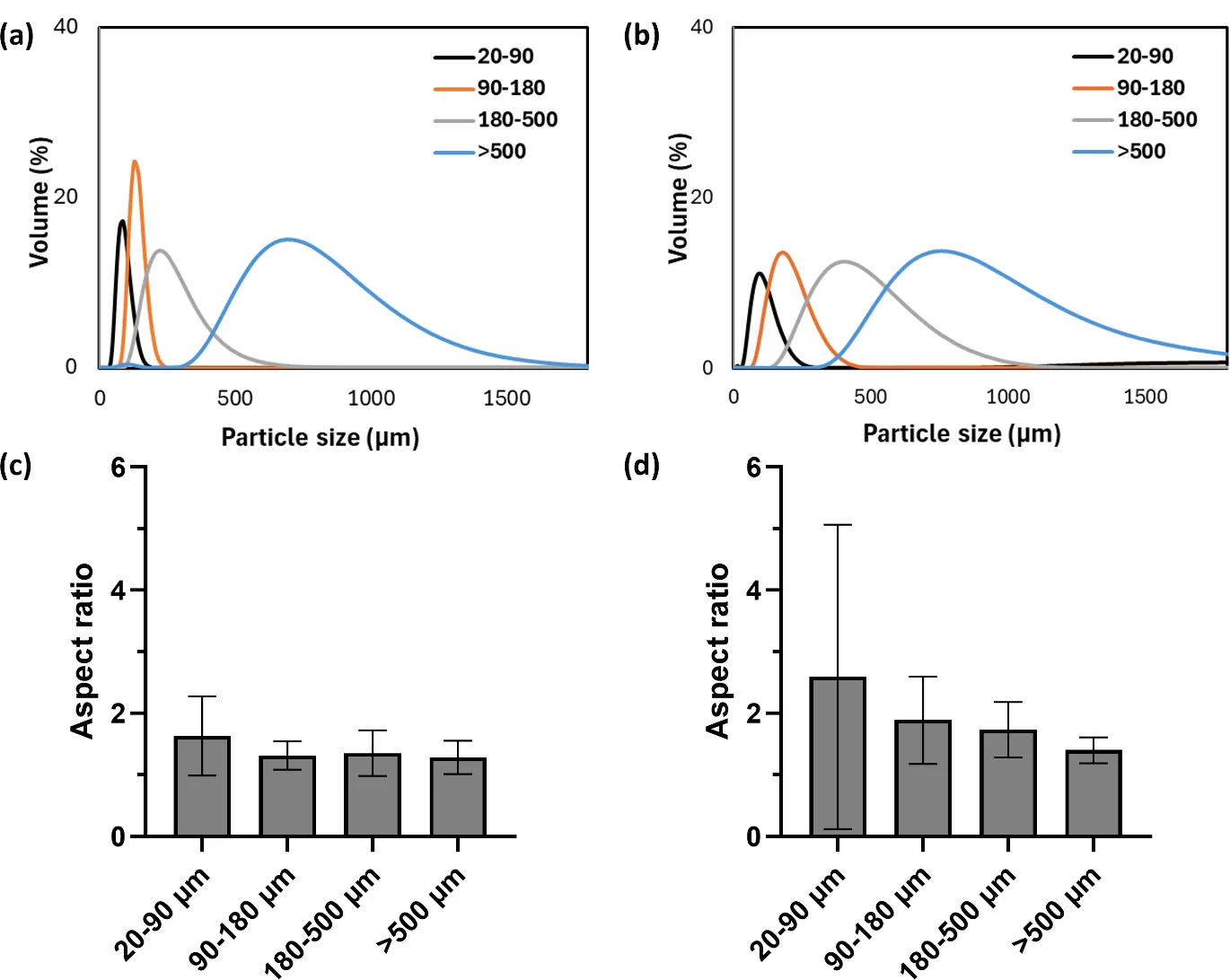
Particle size distribution and aspect ratio of original and ground PVC particles in different size fractions. Particle size distributions of (**a**) original PVC particles and (**b**) ground PVC particles measured by laser diffraction, and aspect ratios of (**c**) original PVC particles and (**d**) ground PVC particles determined from SEM images.

Particle size distribution analysis further confirmed the differences between the two particle types (Fig. 3; Table 1). For the original PVC particles, each sieved fraction showed a relatively narrow and distinct particle size distribution (Fig. 3a), which was also reflected in the percentile diameters. For example, in the 90–180 μm fraction, the original PVC particles had D10, D50, and D90 values of 108, 140, and 183 μm, respectively, with a Sauter mean diameter D[3,2] of 138 μm (Table 1). In contrast, the corresponding ground PVC fraction showed a broader distribution (Fig. 3b) and larger percentile diameters, with D10, D50, and D90 values of 117, 188, and 298 μm, respectively, and a D[3,2] of 176 μm. Similar trends were observed in the larger fractions. For instance, in the 180–500 μm fraction, ground PVC particles had a D[3,2] of 398 μm compared with 236 μm for original PVC particles, and the D90 increased from 403 to 710 μm. In the >500 µm fraction, the D[3,2] values were 630 μm for original PVC and 808 μm for ground PVC, with D90 values of 1,160 and 1,410 μm, respectively. These results indicate that the ground PVC particles had broader distributions and larger apparent particle sizes than the original PVC particles within the same sieved fractions.

**TABLE 1:** Particle size characteristics of original and ground PVC microplastic particles within different size fractions, including Sauter mean diameter (D [3,2]) and percentile diameters (D10, D50, and D90), as measured by laser diffraction

Characteristic	Original PVC particles of size range (μm)				Ground PVC particles of size range (μm)			
	20–90	90–180	180–500	>500	20–90	90–180	180–500	>500
D [3,2] (μm)	84.7	138	236	630	88.3	176	398	808
D10 (μm)	60.9	108	159	484	56.5	117	258	541
D50 (μm)	88.1	140	247	748	101	188	428	843
D90 (μm)	126	183	403	1,160	184	298	710	1,410

The aspect ratio data further supported these morphological differences (Fig. 3c and d). The original PVC particles showed relatively low and consistent aspect ratios across all fractions, confirming their more rounded morphology. In contrast, the ground PVC particles exhibited higher aspect ratios and greater variability, particularly in the smaller fractions. Notably, the 20–90 μm ground PVC fraction showed the highest aspect ratio and the largest variation, indicating the presence of elongated and irregular fragments generated by fracture. Together, these findings confirm that cryogenic grinding altered not only particle surface structure but also particle geometry.

For the subsequent bacterial inactivation experiments, the 90–180 μm fraction was selected to minimize the confounding effect of particle size while preserving the morphological contrast between original and ground PVC. Although both samples were collected within the same sieve range, the ground PVC fraction still displayed a broader size distribution than the corresponding original PVC fraction. This difference likely reflects the irregular and elongated shape of the ground particles. During sonic sieving, elongated particles can reorient and pass through sieve openings along their narrower dimension even when their overall length exceeds the nominal mesh size. In addition, laser diffraction measurements are sensitive to particle geometry and orientation, which can broaden the apparent size distribution of irregular particles (35). In contrast, the more rounded original PVC particles produced more uniform scattering behavior and narrower distributions (36). Nevertheless, by collecting particles that passed through the 180 μm sieve and were retained on the 90 μm sieve, both samples were maintained within a comparable size range while preserving clear morphological differences.

Overall, these results demonstrate successful production of PVC microplastics with distinct and well-characterized morphologies. The combination of cryogenic grinding and sieving provided a practical approach to generate irregular PVC particles while preserving size-defined fractions for comparison with the original rounded particles. This directly addresses the first key contribution of the study by establishing a PVC microplastic model system with a controlled size range but clearly different morphology, thereby providing a useful platform for mechanistic studies on microplastic–microbe interactions and their effects on microbial inactivation.

### Effect of PVC microplastics on bacterial inactivation during thermal and UVC treatments

**Fig 4:**
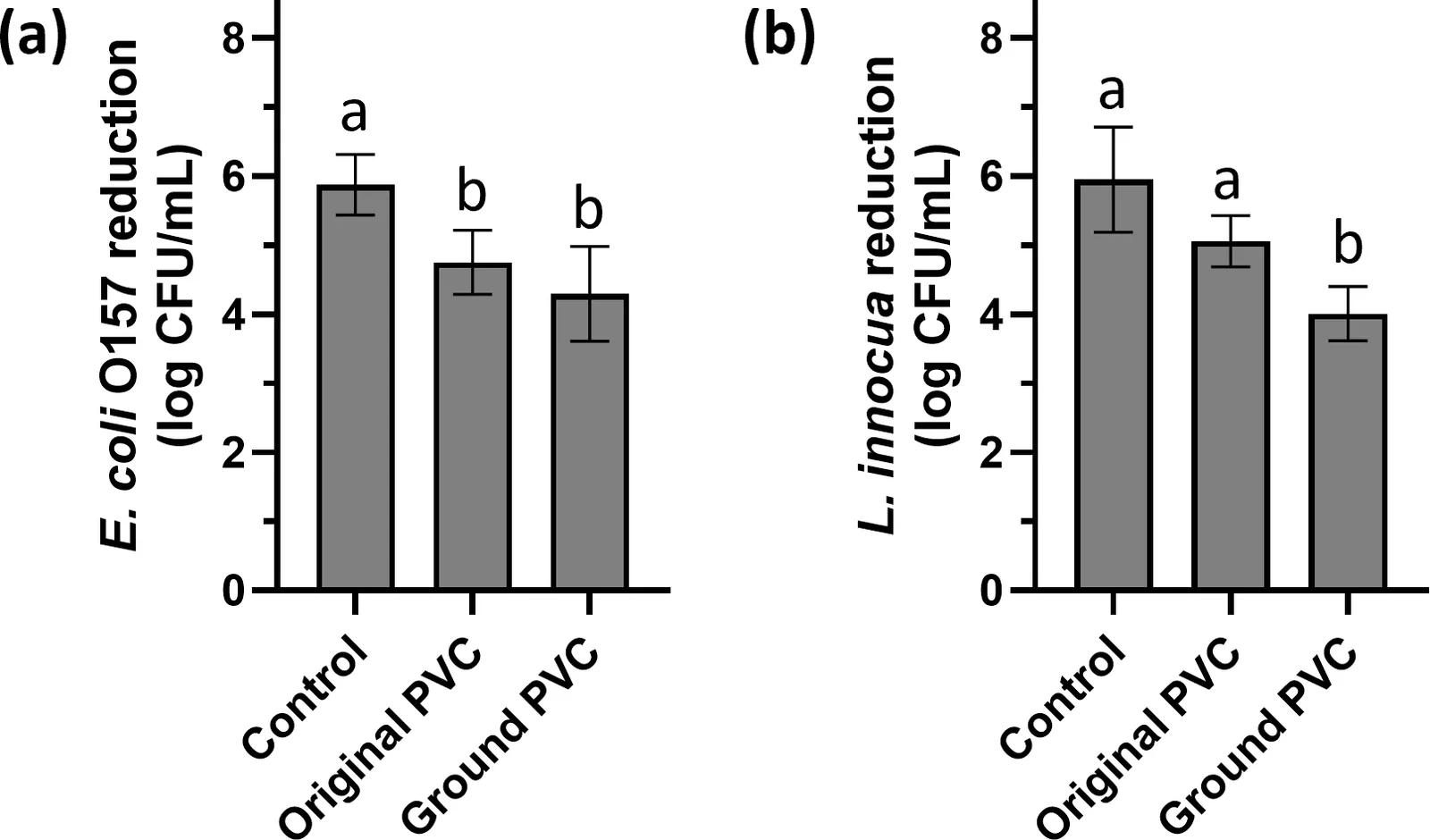
Log reduction of (**a**) *E. coli* O157:H7 and (**b**) *L. innocua* after thermal treatment in the absence and presence of original or ground PVC particles. The treatment conditions were 67°C for 15 s for *E. coli* O157:H7 and 70°C for 15 s for *L. innocua*. Different letters indicate statistical significance at *P* < 0.05, with error bars representing the standard deviations of four independent replicates.

During thermal treatment, the temperature conditions were selected to produce an approximately 5-log reduction in the particle-free controls while accounting for differences in thermal resistance between the two bacteria. Accordingly, *E. coli* O157:H7 was treated at 67°C for 15 s, whereas *L. innocua* was treated at 70°C for 15 s. The presence of PVC microplastics (90–180 μm) reduced bacterial inactivation during both thermal and UVC treatments. Under thermal treatment, the control group, which represents no microplastic treatment, exhibited the highest log reduction, reaching approximately 5.85 log CFU/mL for *E. coli* O157:H7 and 5.95 log CFU/mL for *L. innocua* (Fig. 4). For *E. coli* O157:H7, both original and ground PVC particles significantly reduced thermal inactivation compared with the control, while no significant difference was observed between the two particle types, with log reductions of 4.75 ± 0.46 and 4.30 ± 0.69 log CFU/mL, respectively. For *L. innocua*, ground PVC particles resulted in the lowest log reduction (~4.01 log CFU/mL) and showed a significantly greater protective effect than the control, whereas original PVC particles produced a smaller reduction in inactivation (~5.06 log CFU/mL). Overall, these results suggest that PVC microplastics reduced thermal lethality, with a more pronounced effect observed for the ground particles.

**Fig 5:**
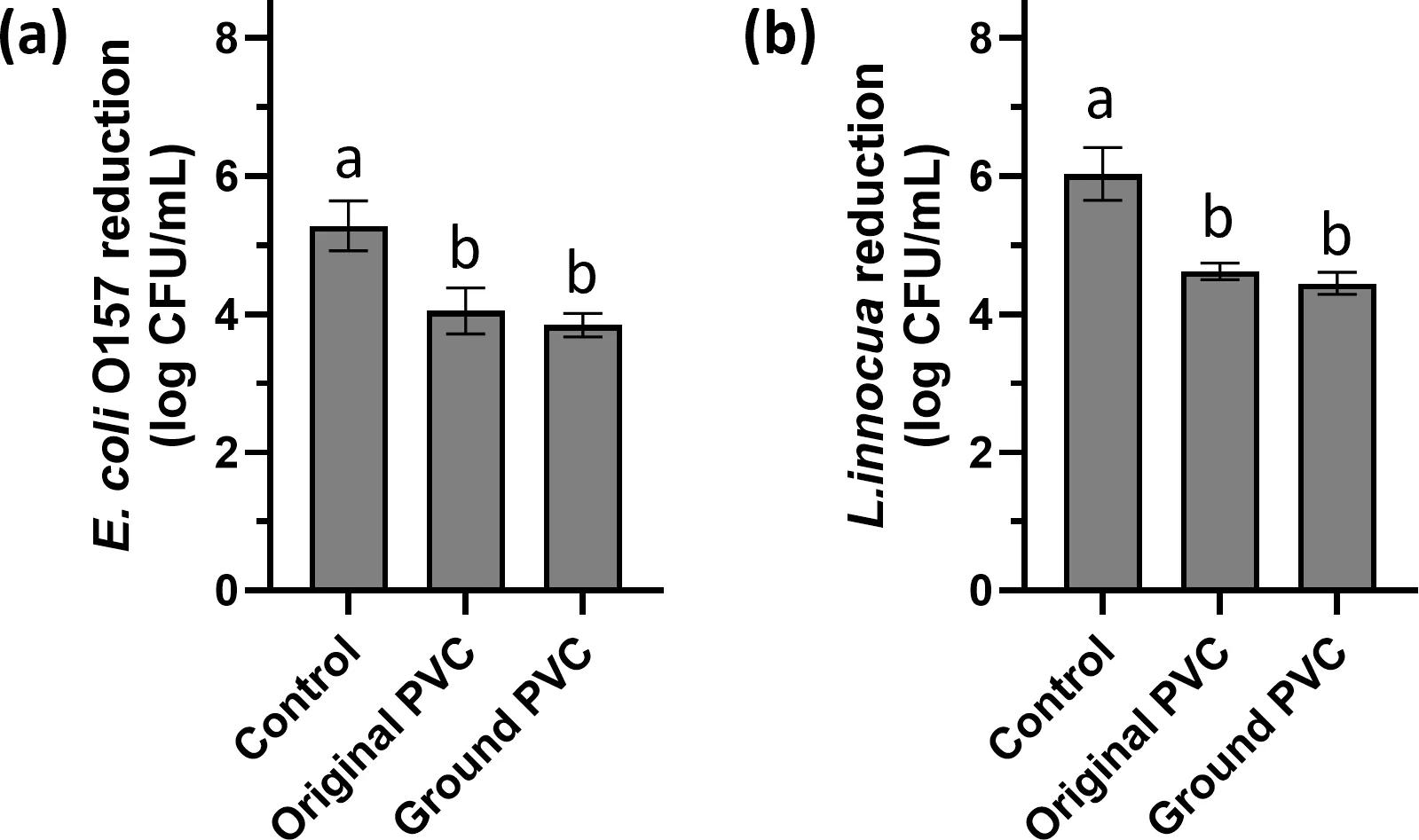
Log reduction of (**a**) *E. coli* O157:H7 and (**b**) *L. innocua* after UVC treatment in the absence and presence of original or ground PVC particles. The UVC irradiance at the sample position was 11 mW/cm^2^, and the exposure conditions were 8 s (88 mJ/cm^2^) for *E. coli* O157:H7 and 18 s (198 mJ/cm^2^) for *L. innocua*. Different letters indicate statistical significance at *P* < 0.05, with error bars representing the standard deviations of four independent replicates.

A similar overall trend was observed during UVC treatment (Fig. 5). In this assay, bacterial cells were exposed to UVC irradiation with an intensity of 11 mW/cm^2^, with exposure times of 8 s for *E. coli* O157:H7 and 18 s for *L. innocua*. In the absence of PVC particles, the control showed the highest log reduction, reaching approximately 5.28 log CFU/mL for *E. coli* O157:H7 and 6.03 log CFU/mL for *L. innocua*. For *E. coli* O157:H7, the presence of original and ground PVC particles significantly reduced the log reduction to approximately 4.04 and 3.84 log CFU/mL, respectively. Similarly, for *L. innocua*, log reduction decreased to approximately 4.62 log CFU/mL in the presence of original PVC particles and 4.45 log CFU/mL in the presence of ground PVC particles. However, no significant difference was observed between the two particle types for either bacterium, indicating that both original and ground PVC particles interfered with UVC inactivation to a similar extent under the tested conditions.

These results show that PVC microplastics reduced the efficacy of both thermal and UVC inactivation, although the underlying mechanisms likely differed between the two treatments. The morphology effect was more apparent during thermal treatment, particularly for *L. innocua*, whereas for UVC treatment, the dominant factor appeared to be the presence of suspended particles themselves. These findings support the second key contribution of this study by demonstrating that PVC microplastics, especially those with irregular morphology, can influence bacterial survival during inactivation in aqueous systems.

### Bacterial–microplastic interactions

To further investigate the mechanisms underlying the reduced inactivation efficiency, bacterial–microplastic interactions were evaluated by confocal microscopy and plate counting methods. Confocal microscopy was used to visualize the spatial distribution of *E. coli* O157:H7 on PVC particle surfaces, while plate counting was used to quantify bacterial attachment after 15-min incubation.

**Fig 6:**
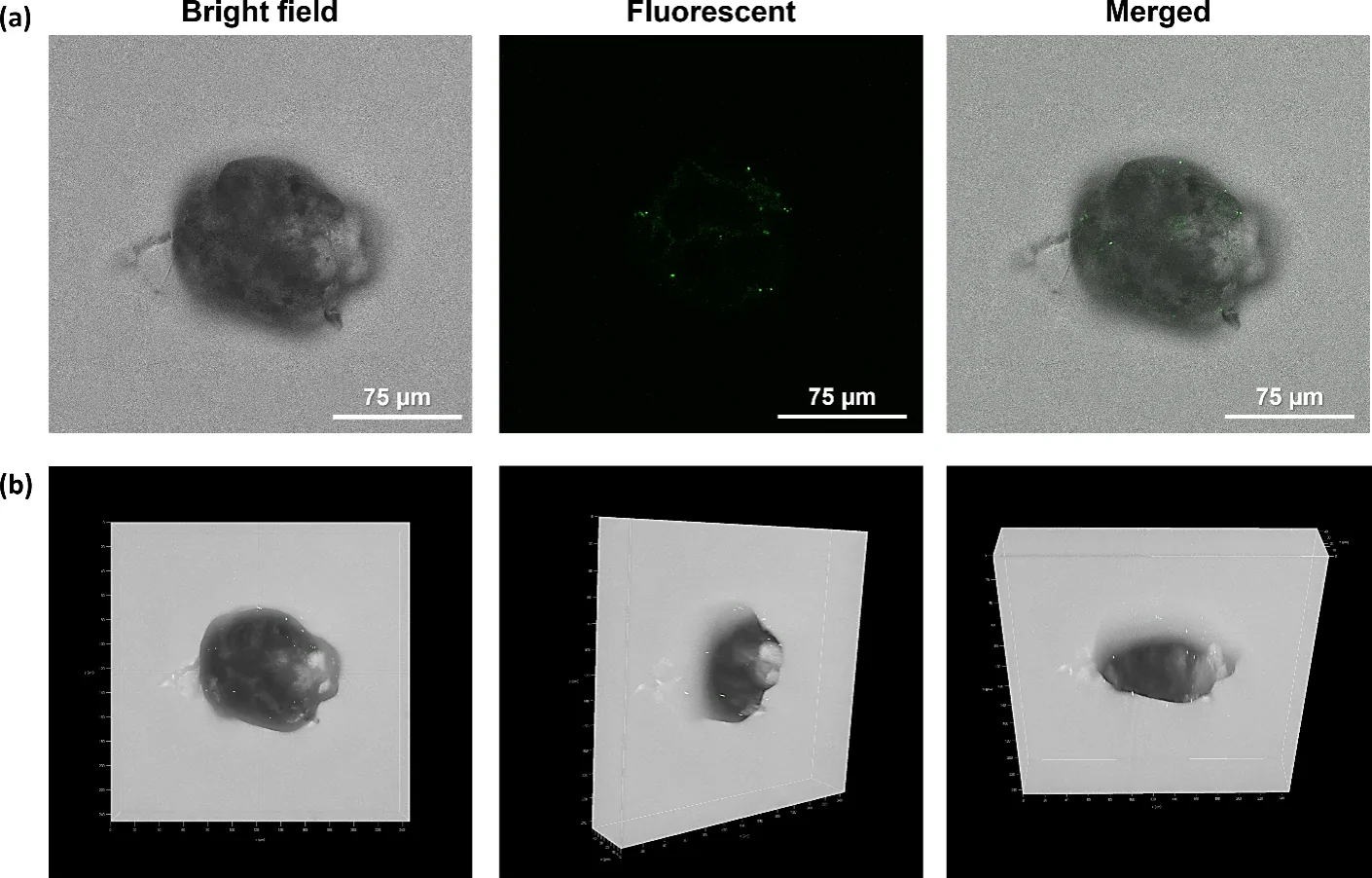
Bacterial–microplastic interaction on an original PVC particle. (**a**) Representative confocal microscopy images showing bacterial distribution on the particle surface in bright-field, fluorescence, and merged views. (**b**) Three-dimensional reconstruction from confocal Z-stack images showing bacterial attachment on the original PVC particle.

**Fig 7:**
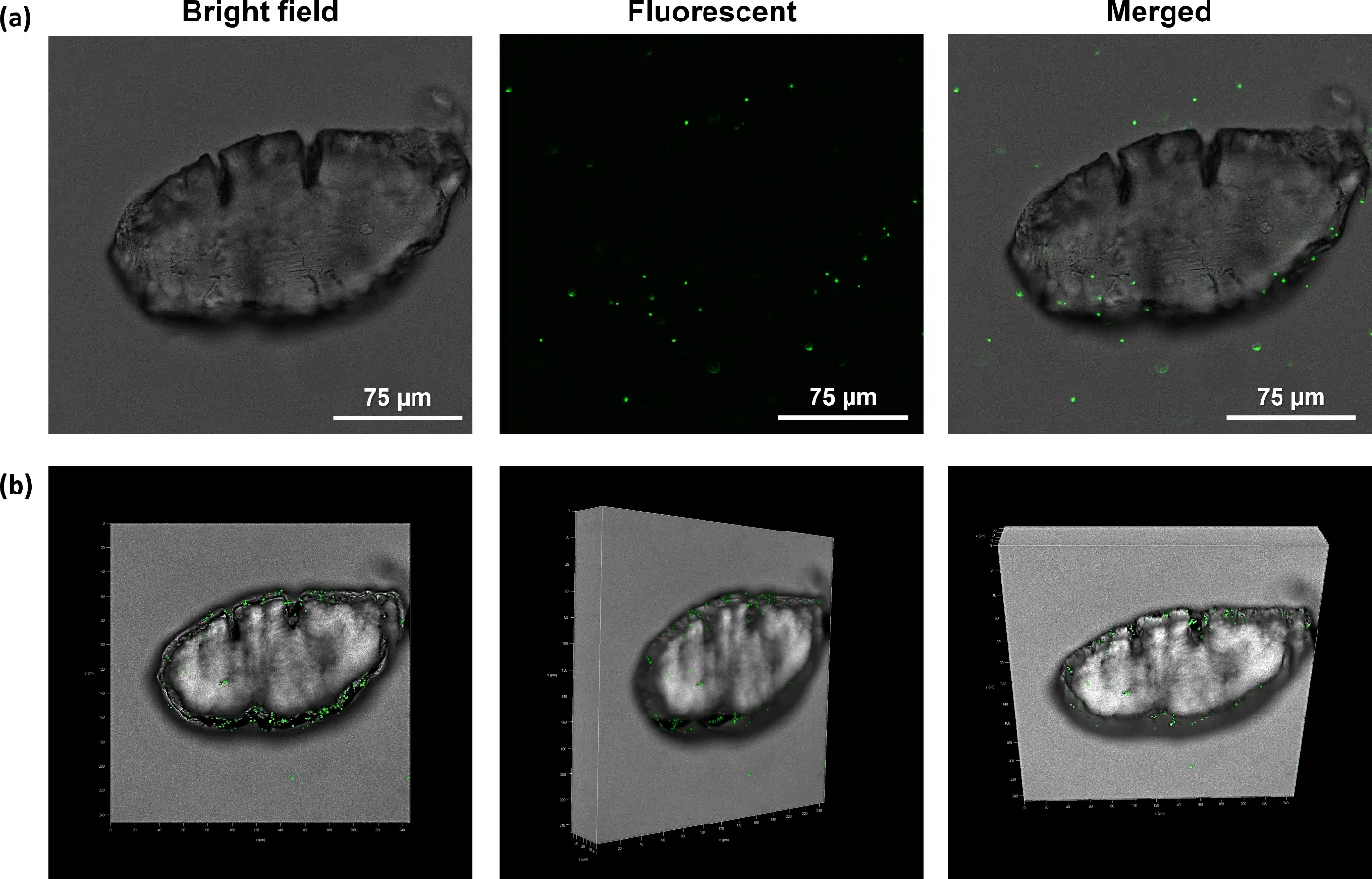
Bacterial–microplastic interaction on a ground PVC particle. (**a**) Representative confocal microscopy images showing *E. coli* O157:H7 distribution on the particle surface in bright-field, fluorescence, and merged views. (**b**) Three-dimensional reconstruction from confocal Z-stack images showing bacterial attachment on the ground PVC particle.

Figures 6 and 7 show confocal microscopy images and three-dimensional reconstructions illustrating the attachment of SYBR Green-stained *E. coli* on original and ground PVC particles. For the original PVC particles, fluorescent signals were detected on localized regions of the particle surface, indicating that bacterial cells attached to the PVC surface rather than remaining uniformly dispersed in the surrounding medium (Fig. 6a). The corresponding three-dimensional reconstruction in Fig. 6b further confirmed that the fluorescent signal was spatially associated with the particle surface. These observations demonstrate that even the relatively smooth and rounded original PVC particles could serve as attachment sites for bacterial cells.

A different attachment pattern was observed for the ground PVC particles (Fig. 7). Compared with the original PVC particles, the ground particles showed more extensive fluorescent signals distributed along the particle edges and surface irregularities. The three-dimensional reconstruction in Fig. 7b also suggested a greater degree of bacterial–particle association on the ground PVC surface. The enhanced attachment of *E. coli* O157:H7 on ground PVC particles can be attributed to their irregular morphology and increased surface roughness, which provide greater surface area and microscale features that promote bacterial adhesion and retention (37).

**Fig 8:**
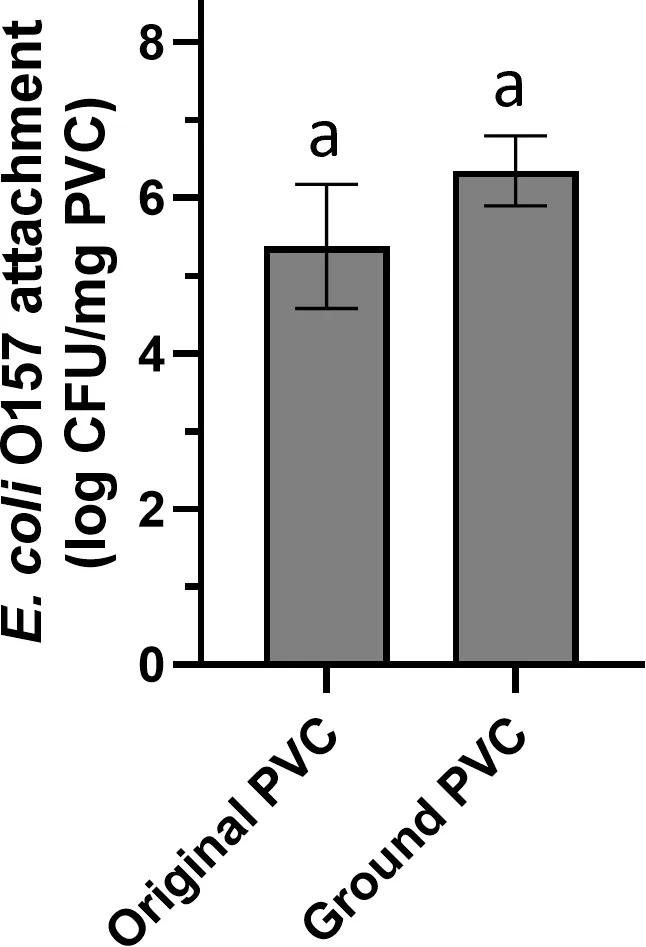
Attachment of *E. coli* O157:H7 to original and ground PVC microplastics after 15-min incubation. Identical letters indicate no significant difference at *P* > 0.05, and error bars represent the standard deviations of four independent replicates.

Quantitative attachment analysis (Fig. 8) further showed that *E. coli* attachment to original and ground PVC particles after 15 min of incubation was 5.38 ± 0.80 log CFU/mg PVC and 6.35 ± 0.45 log CFU/mg PVC, respectively. Although this difference was not statistically significant (*P* > 0.05), the mean attached bacterial population on ground PVC was approximately 9.3-fold higher than on original PVC particles. These results are consistent with the confocal microscopy observations, which showed greater bacterial association with the irregular ground PVC particles.

Overall, these microscopy and plate counting results provide direct evidence that PVC microplastics can act as bacterial carriers in aqueous systems. More importantly, they help explain the reduced bacterial inactivation observed in the presence of PVC particles during thermal and UVC treatment (Fig. 4 and 5). For thermal treatment, bacterial–microplastic association may create localized protection during heating. Compared with the microplastic-free control, the presence of PVC particles resulted in lower log reduction values, suggesting that particle-associated cells were less effectively inactivated. One possible reason is that PVC has lower thermal conductivity than the surrounding aqueous medium (38, 39), which may reduce heat transfer at the particle–cell interface and lead to less uniform thermal exposure of attached bacterial cells (40). This effect appeared stronger for ground PVC particles, which showed lower log reduction values than original PVC particles for both bacterial species, and the confocal results for *E. coli* O157:H7 suggest that stronger bacterial–particle association may be one contributing factor. The greater protective effect of ground PVC is consistent with its more irregular morphology and higher surface heterogeneity, which may promote stronger bacterial–particle association and retention, in line with previous findings that rough or irregular surfaces can enhance microbial attachment (41).

For UVC treatment, bacterial attachment to PVC particle surfaces may also reduce direct light exposure by increasing particle-associated shielding. Although the difference between original and ground PVC particles was less evident under UVC treatment, the confocal images still support the conclusion that bacterial–microplastic interactions contributed to the overall reduction in treatment efficacy.

### Conclusions

This study developed a PVC microplastic model system with distinct particle morphologies and used it to evaluate how particle morphology affects bacterial survival during thermal and UVC inactivation in aqueous systems. Original PVC particles exhibited relatively smooth and rounded morphologies, whereas ground PVC particles showed more irregular shapes, higher aspect ratios, and broader size distributions. The presence of PVC microplastics with the size range of 90–180 µm reduced the inactivation of both *E. coli* O157:H7 and *L. innocua* during both thermal and UVC treatments. During thermal treatment, the ground PVC particles showed a greater protective effect than the original PVC particles, suggesting that irregular morphology enhanced particle-mediated protection, likely through stronger bacterial attachment and reduced local heat transfer. Under UVC treatment, both original and ground PVC particles similarly reduced bacterial inactivation, indicating that particle-associated shielding rather than morphology was the dominant factor under the tested conditions.

Overall, these results demonstrate that PVC microplastics can interfere with microbial inactivation in aqueous systems and that particle morphology plays an important role, particularly during thermal treatment. This work not only provides a useful PVC microplastic model platform with defined morphology and controlled size range but also highlights the need to consider microplastic characteristics when evaluating microbial control processes in food-related environments. Future work might focus on elucidating the physicochemical mechanisms governing interactions between microplastics and bacterial cells, particularly how surface chemistry influences attachment behavior and inactivation resistance.
